# The association between hypoxia-inducible factor-1 α gene G1790A polymorphism and cancer risk: a meta-analysis of 28 case–control studies

**DOI:** 10.1186/1475-2867-14-37

**Published:** 2014-05-01

**Authors:** Yuqiao Zhou, Lin Lin, Yun Wang, Xin Jin, Xin Zhao, Dongjuan Liu, Ting Hu, Lu Jiang, Hongxia Dan, Xin Zeng, Jing Li, Jiayi Wang, Qianming Chen

**Affiliations:** 1 State Key Laboratory of Oral Diseases, West China College of Stomatology, Sichuan University, No.14 the 3rd Section of Renmin South Road, Chengdu, Sichuan 610041, China; 2 Department of Oral Radiology, West China College of Stomatology, Sichuan University, No.14 the 3rd Section of Renmin South Road, Chengdu, Sichuan 610041, China

**Keywords:** Cancer, HIF-1α, Polymorphism, Meta-analysis

## Abstract

**Purpose:**

Hypoxia-inducible factor-1 (HIF-1) is a key transcription factor that regulates the cellular adaptation to hypoxia. HIF-1α gene single nucleotide polymorphisms (SNPs) are implicated to be associated with cancer risks. However, results from the published studies remained inconclusive. The aim of this study is to investigate the relationship of HIF-1α gene G1790A polymorphism with cancer using meta-analysis.

**Methods:**

A comprehensive search in Pubmed, EMBASE and China National Knowledge Infrastructure (CNKI) was conducted to identify all publications on the association between this polymorphism and cancer until December 13, 2013. Odds ratios (OR) with 95% confidence intervals (95% CI) were used to evaluate the strength of this association. Association between lymph node metastasis and G1790A was also investigated.

**Results:**

A total of 5985 cases and 6809 controls in 28 case–control studies were included in this meta-analysis. The A allele of HIF-1α gene G1790A polymorphism was found to be significantly associated with increased cancer risk in four genetic models: AA + AG vs. GG (dominant model OR = 1.85, 95% CI = 1.27-2.69), AA vs. AG + GG (recessive model OR = 5.69, 95% CI = 3.87-8.37), AA vs. GG (homozygote comparison OR = 6.63, 95% CI = 4.49-9.79), and AG vs. GG (heterozygote comparison OR = 2.39, 95% CI = 1.53-3.75). This variant was also significantly associated with higher risks of pancreatic cancer, head and neck cancer, lung cancer and renal cell carcinoma. However, the A allele of G1790A was not significantly associated with increased lymph node metastasis in the dominant model by overall meta-analysis.

**Conclusions:**

Our meta-analysis suggests that the substitution of G with A of HIF-1α gene G1790A polymorphism is a risk factor of cancer, especially for pancreatic cancer, lung cancer, renal cell carcinoma and head and neck cancer. The association is significant in Asian, Caucasian population and public based control subgroups. However, it’s not associated with risk of lymph node metastasis.

## Introduction

Cancer is a multifactorial disease involving interactions between inherited and environmental factors [1]. Hypoxia is one of the fundamentally important characteristics of solid cancer [2]. It triggers a cascade of molecular events including angiogenesis and involving cell-cycle control proteins, which are closely associated with tumor growth, metastasis and poor prognosis. These responses to hypoxia are highly dependent on the activation of hypoxia inducible factor-1-alpha (HIF-1α).

Hypoxia-inducible factor-1 (HIF-1) is a key transcription factor that regulates the cellular adaptation to hypoxia. HIF-1 trans-activates a large number of genes that are involved in cellular processes, such as glucose uptake and metabolism, angiogenesis, cell proliferation, differentiation and apoptosis [3,4]. HIF-1 is a heterodimeric transcription factor that consists of α and β subunits. Β subunit is constitutively expressed while the expession of HIF-1α is regulated by the oxygen level [5]. Under normoxic conditions, HIF-1α is degraded due to targeted ubiquitination and degradation by the proteasome. This process is mediated by direct binding of von Hippel–Lindau tumor suppressor protein (pVHL), a component of the E3 ubiquitin–protein ligase complex, with the minimal N-terminal transactivation domain (N-TAD), which is located within the oxygen-dependent degradation domain of HIF-1α. On the contrary, in hypoxic conditions, the degradation of HIF-1α is suppressed and the expression of HIF-1α increases in the cell. Over-expression of HIF-1α has been reported in many types of cancer, including lung, prostate, breast, colon and rectum carcinoma, and in regional or distant metastases, implying that it may play a vital role in tumor progression [6-11].

A number of polymorphisms and mutations have been identified within HIF-1α gene, among them, G1790A (rs11549467), which results in alanine to threonine amino acid substitutions has been found recently. It is located within the oxygen-dependent degradation domain of the HIF-1α gene. This polymorphic variant has been shown to cause an increased trans-activation capacity of HIF-1α under hypoxic conditions *in vitro*[12]. The possible association between HIF-1α SNPs and cancer risk has been studied by several investigators, but the results were inconclusive or even contradictory [7,9,11,13-20]. Tongfeng Zhao et al. [21] conducted a meta-analysis about HIF-1α polymorphisms and cancer risk in 2009, however, the sample size was limited (2058 cancer cases and 3062 controls) and studies published in the past 3 years were not included. In order to obtain a more comprehensive knowledge of the association between HIF-1α G1790A polymorphism and cancer risk, a meta-analysis on eligible case–control studies was conducted.

## Method

### Literature search and data extraction

A comprehensive literature search in PubMed, Embase and China National Knowledge Infrastructure (CNKI) database was carried out by two reviewers independently to identify publications evaluating the association of cancer risk and HIF-1α G1790A polymorphism (last update: December 13, 2013). The search terms used were as follows: (cancer or carcinoma) and (Hypoxia-inducible factor-1α or HIF-1α) and (Polymorphism or mutation or variant). Selection criteria of an eligible study were: (a) investigation of the polymorphism G1790A of HIF-1α and cancer risk; (b) use of a case–control design based on unrelated individuals and (c) sufficient genotype distributions for cases and controls so that an odds ratio (OR) with 95% confidence interval (CI) could be estimated. If more than one article was published using the same patient population, only the latest or the study with largest sample size would be used in this meta-analysis.

Data including publication date, first author, original country, ethnicity, gender and cancer site (those cancer sites only exist in one article were divided into “other site group”), total number of casea and controla, source of control (population-based or hospital-based) and genotyping method were independently extracted by two investigators and conformity on all items was reached through consultation. Articles containing data on lymph node metastasis were also extracted. Patients were divided into “lymph node metastasis positive” group and “lymph node metastasis negative” group [8-10,16,22-24].

### Statistical study

Crude odds ratios (ORs) and 95% confidence intervals (95% CIs) were used to estimate the strength of association between the G1790A and cancer risk or lymph node metastasis and they were determined by Z-test. The pooled ORs were estimated for the genetic models including dominant model (AA + AG vs. GG), recessive model (AA vs. AG + GG), homozygote comparison (AA vs. GG) and heterozygote comparison (AG vs. GG). The models were selected based on the following assumptions:

1. Dominant model: The heterozygous (AG) and homozygous (AA) genotypes have similar risk as a single copy of A is sufficient to alter the risk. Hence these two genotypes are combined and compared with the homozygous GG.

2. Recessive model: The AG and GG genotypes have the same effect. Hence to modify two copies of A allele is essential. Therefore this model compares the combination of AG + GG with the homozygous genotype variant allele AA.

3. Homozygote/Heterozygote comparison: Ignoring the effect of heterozygotes/homozygotes.

The significance of OR was analyzed by Z test, *P* < 0.05 was considered statistically significant. As most articles only used (AA + AG vs. GG) model for lymph node analysis, only this genetic model (AA + AG vs.GG) was used in the lymph node metastasis analysis.

In addition to the comparison between cases and controls, subgroup analyses were also performed according to cancer site, ethnicity, source of controls and gender.

Heterogeneity was evaluated by a chi square-based Q statistic, and statistical significance was assumed for *P* value less than 0.05. A fixed-effect model was used when *P* heterogeneity was more than 0.05, otherwise a random effect model was used. Sensitivity analysis was performed to assess the stability of the current analysis. Studies which didn’t follow Hardy–Weinberg equilibrium (HWE) were excluded [25].

Publication bias was examined visually by the Begg’s funnel plot and the degree of asymmetry was tested by Egger’s test.

All of the statistical tests were performed by STATA11.0.

## Results

### Characteristics of studies included

#### ***Basic characteristics of studies***

**Figure 1:**
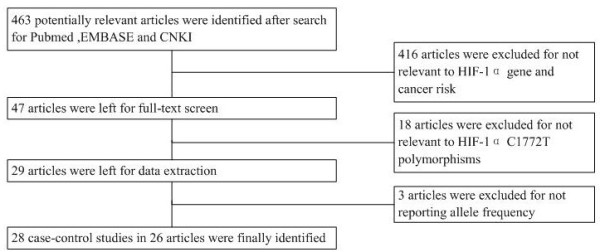
Flow chart of the process of selecting related publications.

463 relevant publications were identified after initial screening based on our search criteria (last updated on December 13, 2013). After reading the titles and abstracts, 47 articles were subjected to further examination. 18 articles were excluded for no relevance to G1790A. 3 articles were excluded as allele frequency was not reported (Figure 1). Finally, a total of 28 case–control studies from 26 articles (Table 1) with 5985 cases and 6809 controls in total were included in the pooled analysis. Out of those 28 studies, 6 studies focused on head and neck cancer, 3 on prostate cancer, 4 on breast cancer, 3 on lung cancer, 3 on renal cell carcinoma, 2 on pancreatic cancer and 7 on other types of cancers such as gastric, hepatocellular and colorectal cancer. Among the 26 articles, 1 article provided data on three types of cancer (cervical cancer, endometrial cancer and ovarian cancer) [7]. Each type of cancer in one article was treated as an individual study in the meta-analysis. The ethnicities studied included Caucasian (13 articles), Asian (13 articles), Brazilian (1 article) and mixed population (1 article). The gender studied were female (8 articles), male (17 articles) and mixed (3 articles). 7 articles provided data on lymph node metastasis, 1511 negative cases and 491 positive cases were included.

**Table 1:** Characteristics of populations and cancer types of the studies included in the meta-analysis

**Author**	**Year**	**Country**	**Ethnicity**	**Cancer site**	**Case–control**	**Gender**^**a**^	**Soure of control**	**Genotyping method**	**HWE**
**Ribeiro AL**	2013	Portugal	Caucasian	Breast cancer	96/74	Female	HB	PCR-RFLP	0.00
**Alves LR**	2012	Brazil	Brazilian	Head and neck cancer	88/40	Mixed	PB	PCR-RFLP	0.69
**Li P**	2012	China	Asian	Prostate cancer	662/716	Male	PB	TaqMan	0.55
**Mera-Menendez F**	2012	Spain	Caucasian	Head and neck cancer	121/154	Mixed	PB	PCR–RFLP	0.69
**Ruiz-Tovar J**	2012	Spain	Caucasian	Pancreatic cancer	59/159	Mixed	PB	PCR-RFLP	0.68
**Kuo WH**	2012	China	Asian	Lung cancer	285/300	Mixed	PB	PCR–RFLP	0.15
**Qin C**	2012	China	Asian	Renal cell carcinoma	620/623	Mixed	PB	PCR-RFLP	0.42
**Wang X**	2011	China	Asian	Pancreatic cancer	263/271	Mixed	PB	PCR-RFLP	0.49
**Putra AC**	2011	Japan	Asian	Lung cancer	83/110	Mixed	PB	PCR	0.65
**Kim YH**	2011	Korea	Asian	Other cancer	199/215	Female	PB	PCR-RFLP	0.14
**Shieh TM**	2010	China	Asian	Head and neck cancer	305/96	Mixed	PB	PCR-RFLP	0.71
**Hsiao PC**	2010	China	Asian	Other cancer	102/347	Mixed	HB	PCR-RFLP	0.70
**Chen MK**	2009	China	Asian	Head and neck cancer	174/347	Mixed	PB	PCR-RFLP	0.70
**Naidu R**	2009	Malaysia	Asian	Breast cancer	410/275	Female	PB	PCR-RFLP	0.90
**Konac E**	2009	Turkey	Caucasian	Lung cancer	141/156	Mixed	HB	PCR-RFLP	0.94
**Li K**	2009	China	Asian	Other cancer	87/106	Mixed	PB	PCR	0.76
**Munoz-Guerra MF**	2009	Spain	Caucasian	Head and neck cancer	74/139	Mixed	PB	PCR-RFLP	0.69
**Kim HO**	2008	Korea	Asian	Breast cancer	90/102	Female	PB	PCR	0.06
**Apaydin I**	2008	Turkey	Caucasian	Breast cancer	102/102	Female	PB	PCR-RFLP	0.84
**Li H**	2007	USA	Mixed	Prostate cancer	1072/1271	Male	PB	PCR-RFLP	0.81
**Orr-Urtreger A**	2007	Israel	Caucasian	Prostate cancer	402/300	Male	PB	PCR-RFLP	0.95
**Fransen K**	2006	Sweden	Caucasian	Other cancer	198/258	Mixed	PB	PCR-RFLP	0.77
**Konac E-O**^**b**^	2007	Turkey	Caucasian	Other cancer	49/107	Female	PB	PCR-RFLP	0.00
**Konac E-C**^**c**^	2007	Turkey	Caucasian	Other cancer	32/107	Female	PB	PCR-RFLP	0.00
**Konac E-E**^**d**^	2007	Turkey	Caucasian	Other cancer	21/107	Female	PB	PCR-RFLP	0.00
**Ollerenshaw M**	2004	UK	Caucasian	Renal cell carcinoma	160/288	Mixed	PB	PCR-RFLP	0.00
**Tanimoto K**	2003	Japan	Asian	Head and neck cancer	55/110	Mixed	PB	PCR-RFLP	0.65
**Clifford SC**	2001	UK	Caucasian	Renal cell carcinoma	35/143	Mixed	HB	PCR-RFLP	0.00

^a^Mixed means samples contain both female and male; PB, public based; HB, hospital based; HWE, Hardy-Weinberg equilibrium; PCR, polymerase chain reaction; RFLP, restriction fragment length polymorphism; TaqMan, TaqMan SNP Genotyping Assays.

^**bcd**^The article of Konac E provided data on three kinds of cancers (cervical cancer, endometrial cancer and ovarian cancer), Konac E-O stands for ovarian cancer; Konac E-C stands for cervical cancer; Konac E-E stands for endometrial cancer.

### Characteristics of allele average frequency

**Figure 2:**
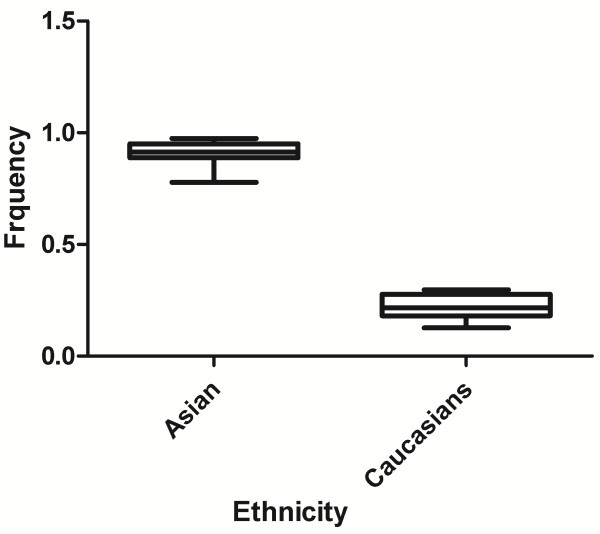
A allele frequency of HIF-1α G1790A among control subjects stratified by ethnicity.

In the control group, no significant difference of the average A allele frequency of HIF-1α G1790A was detected between Asian population (0.040) and Caucasian population (0.045) (*P* = 0.7, t-test) (Figure 2).

### Quantitative data synthesis

#### ***G1790A polymorphisms and cancer: a meta-analysis of 28 case–control studies***

**Figure 3:**
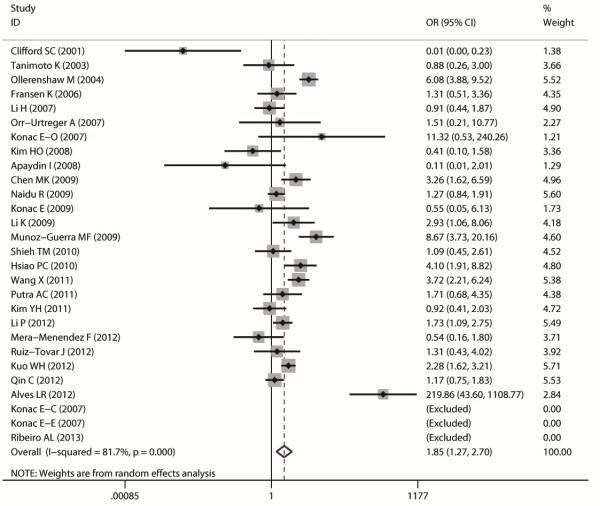
**Forest plot of cancer risk associated with G1790A (AA + AG VS GG).** The squares and horizontal lines represent OR and 95% CI, respectively. The area of the squares indicates the study-specific weight.

**Figure 4:**
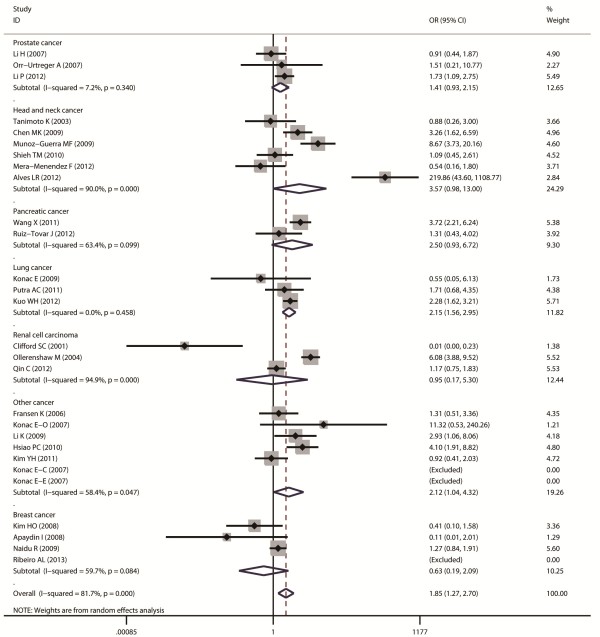
Forest plot of cancer risk associated with G1790A (AA + AG VS GG) stratified by cancer site.

**Figure 5:**
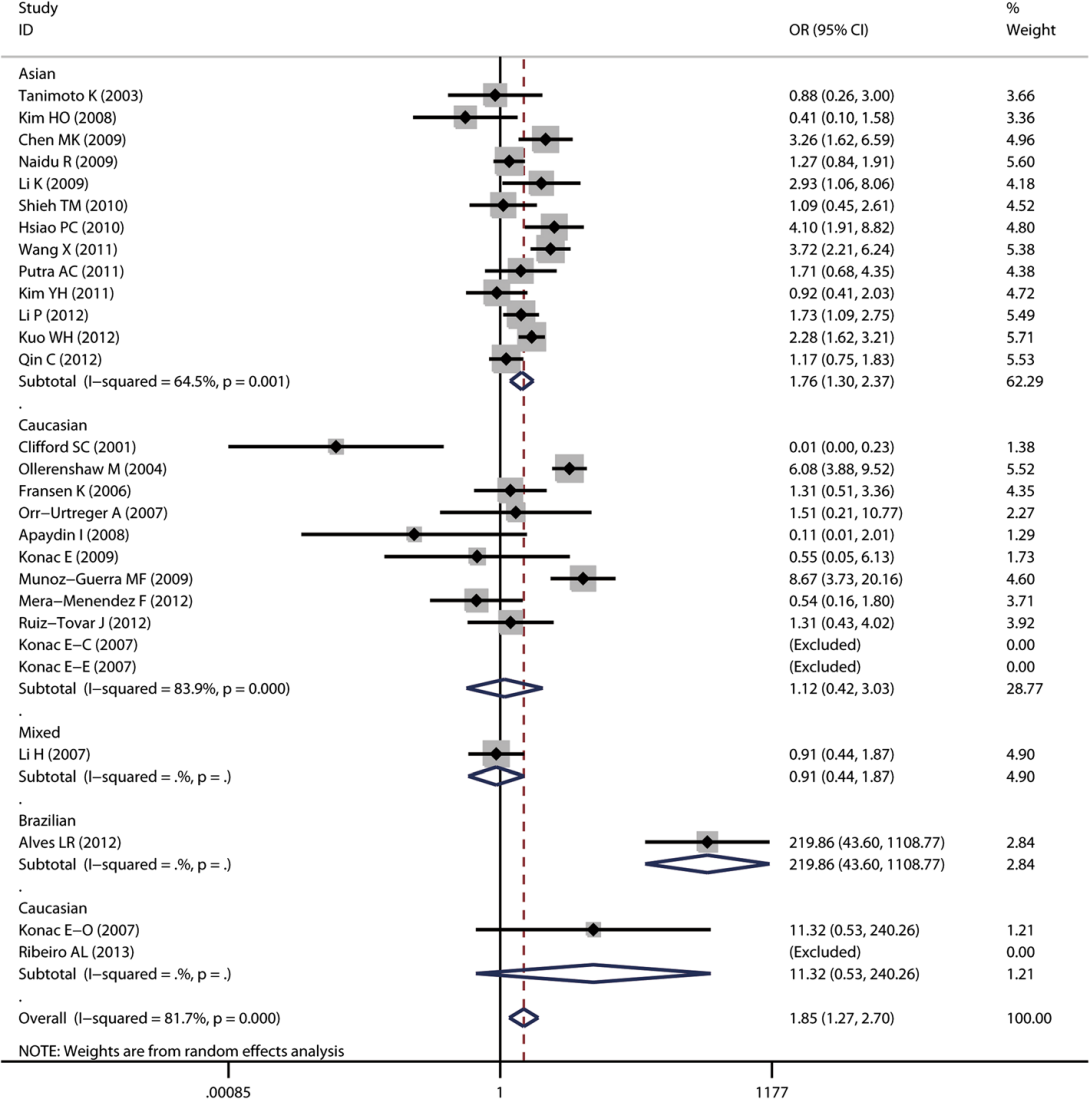
Forest plot of cancer risk associated with G1790A (AA + AG VS GG) stratified by ethnicity.

**Figure 6:**
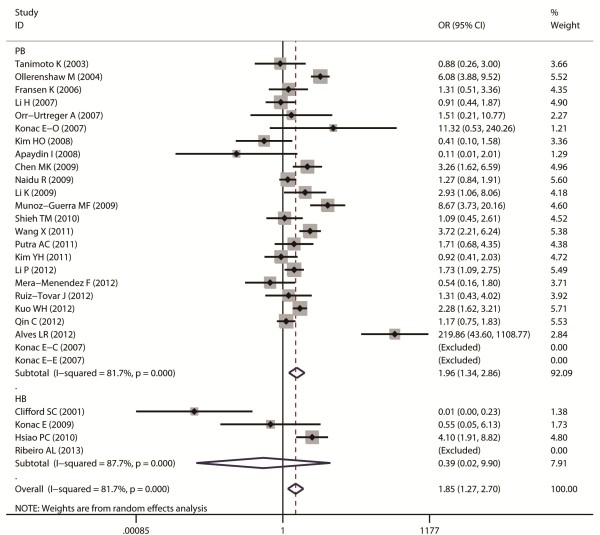
Forest plot of cancer risk associated with G1790A (AA + AG VS GG) stratified by source of control subjects.

**Figure 7:**
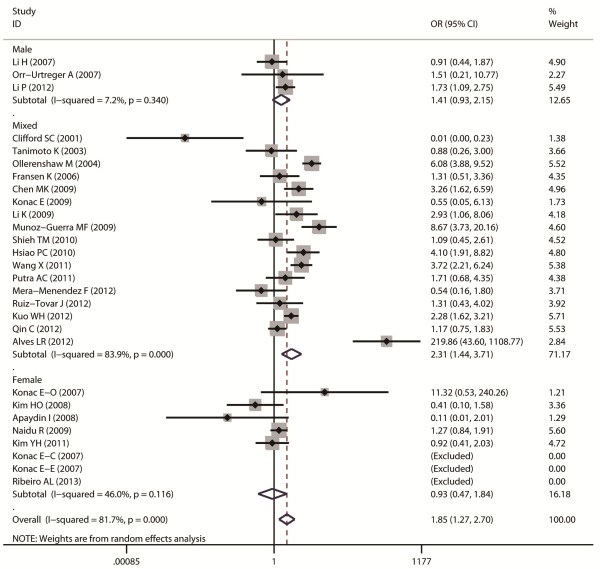
Forest plot of cancer risk associated with G1790A (AA + AG VS GG) stratified by gender.

Aggregated ORs and heterogeneity test results for the association between G1790A and cancer risk were shown in Table 2. The forest plots of cancer risk associated with G1790A (AA + AG vs. GG) were shown in Figures 3, 4, 5, 6 and 7.

**Table 2:** Associations between the G1790A polymorphism and cancer risk

**Varibles**	**AA + AG VS GG**			**AA VS AG + GG**			**AA VS GG**			**AG VS GG**		
	**n**^**a**^	**OR (95% CI)**	***P***^**b**^	**n**^**a**^	**OR (95% CI)**	***P***^**b**^	**n**^**a**^	**OR (95% CI)**	***P***^**b**^	**n**^**a**^	**OR (95% CI)**	***P***^**b**^
**Overall**	25	*1.85 (1.27-2.69 )*	0	26	*5.69 (3.87-8.37)*	0	12	*6.63 (4.49-9.79)*	0	11	*2.39 (1.53-3.75)*	0
**Overall for HWE**^**c**^	22	*1.82 (1.27-2.59)*	0	10	*7.01 (4.42-11.09)*	0	10	*7.75 (4.87-12.34)*	0	10	*3.4 (2–5.8)*	0
**cancer cite**												
**Prostate cancer**	3	1.41 (0.93-2.14)	0.1	1	3.24 (0.13-79.9)	0.47	1	3.34 (0.13-82.30)	0.46	1	1.98 (0.07-50.4)	0.67
**Head and neck cancer**	6	3.57 (0.98-12.99)	0.05	3	*58 (1.75-1924.88)*	0.02	3	*101.38 (22.09-65.29)*	0	3	*12.53 (2.42-64.76)*	0
**Pancreatic cancer**	2	2.5 ( 0.93-6.72)	0.06	1	18.8 (0.96-371.55)	0.05	1	18.3 (0.93-360.19)	0.05	1	*29.4 (1.12-772.37)*	0.04
**Lung cancer**	3	*2.14 ( 1.56-2.95)*	0	2	*4.5 (2.3-8.81)*	0	2	*5.42 (2.74-10.7)*	0	2	*3.02 (1.48-6.16)*	0
**Renal cell carcinoma**	3	0.94 ( 0.16-5.29)	0.95	2	*2.69 (1.20-6.03)*	0.01	2	*3.71 (1.72-7.99)*	0.46	1	0.81 (0.33-2)	0.65
**Other cancer**	5	*2.11 ( 1.03-4.32)*	0.45	1	0.35 (0.01-8.80)	0.53	1	0.35 (0.01-8.8)	0.52	1	0.36 (0.01-9.68)	0.54
**Breast cancer**	3	0.63 ( 0.19-2.08)	0	2	1.44 (0.34-6.08)	0.62	2	1.43 (0.37-5.44)	0.59	2	1.45 (0.34-6.17)	0.61
**Subgroup by ethnicity**												
**Asian**	13	*1.75 (1.3-2.37)*	0	7	*3.37 (1.94-5.86)*	0	7	*3.82 (2.19-6.66)*	0	7	*2.41 (1.31-4.43)*	0
**Caucasian**	9	*1.12 (0.41-3.03)*	0.81	4	*3.74 (1.85-7.54)*	0	4	*5.05 (2.53-10.07)*	0	3	*2.66 (0.33-21.23)*	0.35
**control source**												
**PB**	22	*1.95 (1.33-2.85)*	0	11	*5.9 (4.0-8.8)*	0	11	*7.2 (4.79-10.82)*	0	11	*2.39 (1.53-3.75)*	0
**HB**	3	0.39 (0.01-9.90)	0.56	1	0.88 (0.04-16.82) ^c^	0.93	1	0.44 (0.02-8.53)	0.59	0	No statistics	
**Gender**												
**Female**	5	0.92 (0.46-1.84)	0.82	3	1.11 (0.34-3.66)	0.85	3	1.13 (0.34-3.7)	0.83	3	1.13 (0.31-4.02)	0.84
**Male**	3	1.41 (0.93-2.14)	0.1	1	3.24 (0.13-79.9)	0.47	1	3.34 (0.13-82.3)	0.46	1	1.98 (0.07-50.4)	0.67

^a^Number of comparisons.

^b^*P* value of Q-test for heterogeneity test.

^c^Data after excluding those studies’ controls not in Hardy-Weinberg equilibrium.

Overall, A allele of G1790A was found to be significantly associated with increased cancer risk in four genetic models: AA + AG vs. GG (dominant model OR = 1.85, 95% CI = 1.27-2.69), AA vs. AG + GG (recessive model OR = 5.69, 95% CI = 3.87-8.37), AA vs. GG (homozygote comparison OR = 6.63, 95% CI = 4.49-9.79), and AG vs. GG (heterozygote comparison OR = 2.39, 95% CI = 1.53 -3.75).

In cancer-site subgroup analysis, significant association with cancer risk was found in the following genetic models: AA vs.AG in pancreatic cancer (OR = 29.4, 95% CI = 1.12-772.37); AA + AG vs. GG (OR = 2.14, 95% CI = 1.56-2.95), AA vs. GG + AG (OR = 4.5, 95% CI = 2.3-8.81) and AA vs. GG (OR = 5.42, 95% CI = 2.74-10.7) in lung cancer; AA vs. GG + AG (OR = 58, 95% CI = 1.75-1924.88), AA vs. GG (OR = 101.38, 95% CI = 22.09-65.29 ) and AG vs. GG (OR = 12.53, 95% CI = 2.42-64.76) in head and neck cancer; AA vs. GG + AG (OR = 2.69, 95% CI = 1.2 -6.03) and AA vs.GG (OR = 3.71, 95% CI = 1.72-7.99) in renal cell carcinoma.

As for ethnicity subgroup analysis, significant association was detected in Asian population in all four genetic models: AA + AG vs. GG (OR = 1.75, 95% CI = 1.3-2.37), AA vs. AG + GG (OR = 3.37, 95% CI = 1.94-5.86), AA vs. GG (OR = 3.82, 95% CI = 2.19-6.66), and AG vs. GG (OR = 2.41, 95% CI = 1.31-4.43). However, in Caucasian subgroup, significant increased risk was only found in the following models: AA vs. GG + AG (OR = 3.74, 95% CI = 1.85-7.54) and AA vs. GG (OR = 5.05, 95% CI = 2.53-10.07).

When stratified by control sources, significant association was found among public based control source in these models: AA + AG vs. GG (OR = 1.91, 95% CI = 1.3-2.8), AA vs. AG + GG (OR = 5.9, 95% CI = 3.97-8.75), AA vs. GG (OR = 7.2, 95% CI = 4.79-10.82), and AG vs. GG (OR = 2.39, 95% CI = 1.53-3.7). However, no significant association was found among hospital based control source.

#### ***G1790A polymorphisms and lymph node metastasis: a meta-analysis of 7 case–control studies***

As for lymph node metastasis analysis, no significant association between A allele of G1790A and increased lymph node metastasis was found for AA + AG vs.GG (OR = 1.56, 95% CI = 0.99-2.46).

When stratified by ethnicity and control source, significant higher risk of lymph node metastasis was found in Caucasian (OR = 14.28, 95% CI = 1.74-117.17), Asian (OR = 1.39, 95% CI = 1.01-1.92) and public based population (OR = 1.62, 95% CI = 1.01-2.61) for AA + AG vs.GG (Table 3).

**Table 3:** Associations between the G1790A polymorphism and lymph node metastasis risk

**Varibles**	**n**^**a**^	**AA + AG VS GG**	
		**OR (95% CI)**	***P***^**b**^
**Overall**	7	1.56 (0.99-2.46)	0.05
**PB**	6	*1.62 (1.01-2.61)*	0
**Asian**	6	*1.39 (1.01-1.92)*	0.04
**Caucasian**	1	*14.28 (1.74-117.17)*	0.01

^a^Number of comparisons.

^b^*P* value of Q-test for heterogeneity test.

### Heterogeneity analysis and sensitivity analysis

Significant heterogeneity was found in overall comparisons in four genetic models: dominant model *P* < 0.01, recessive model *P* < 0.01, homozygote comparison *P* < 0.01 and heterozygote comparison *P* < 0.05. Although the genotype distribution in the control group of 6 studies did not follow HWE, the pooled ORs only altered a little after we performed the sensitivity analysis, and the conclusion didn’t change (Table 2).

### Publication bias

**Figure 8:**
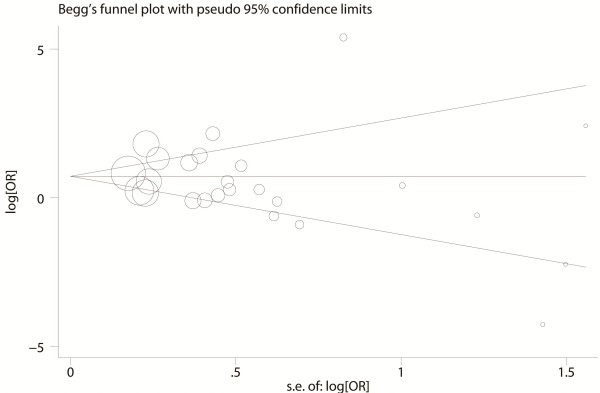
**Funnel Plot of publication bias of the G1790A Polymorphism.** Each point represents a single study for the indicated association. Log (OR): natural logarithm of OR. Horizontal line: mean effect size.

The shape of the funnel plots did not reveal any evidence of obvious asymmetry (Figure 8), suggesting that there was no obvious publication bias. Egger’s test showed no significant publication bias in this meta-analysis (t = 0.77, *P* = 0.45 for AA + AG vs. GG).

## Discussion

HIF-1 is a key transcription factor that regulates cellular reaction to hypoxia and is over-expressed in most solid tumors in response to low oxygen concentrations [12]. It has the ability to influence metabolic reprogramming, angiogenesis, metastasis and over-expression of HIF-1α has been reported in many types of cancers as well as in regional or distant metastatic lesions [22,26].

Several polymorphisms in HIF-1 gene have been suggested to be related with individual’s predisposition to cancer [7,27,28]. Among them, G1790A has been recently identified [27]. The possible association between G1790A and cancer risk has been extensively studied, but the results are inconclusive. One possible reason is that the rareness of A allele in both case and control populations [29] allows only a small number of A carriers to be analyzed in many studies. In order to get a more precise conclusion, a comprehensive meta-analysis including 28 case–control studies was performed.

In the current study, we summarized the latest data on the association between G1790A of HIF-1α gene polymorphism and cancer risk. Subgroup analysis by cancer site, ethnicity, source of controls and gender were also performed, based on four genetic models.

Overall meta-analysis showed that the A allele of G1790A was significantly associated with increased cancer risk in all four genetic models. One possible mechanism is that G1790A SNP results in amino acid change from alanine 588 to threonine within the Oxygen dependent degradation (ODD) domain of the HIF-1α gene. Such amino acid change in this critical regulatory domain may enhance the transcriptional activity of HIF-1α gene [12]. It may also increase the stability of HIF-1α protein and promote the binding ability of HIF-1α protein with the accessory proteins involved the expression of downstream target genes. The protein products of these target genes play crucial roles in the acute and chronic adaptation of cells to oxygen deficiency, including erythropoiesis, glycolysis, promotion of cell survival, inhibition of apoptosis, inhibition of cell differentiation and angiogenesis, all of which are critical in tumor formation, invasion and metastasis [27]. Further studies are required to address this question.

When stratified by cancer site, significant association between A allele of G1790A and increased cancer risk was observed in pancreatic cancer, lung cancer, renal cell carcinoma and head and neck cancer, but not in breast cancer, prostate cancer and other cancers. As cancer of different sites are exposed to different micro-environmental factors that can regulate or influence the gene expression profiles, gene-environment interaction may be different in these cancers. Furthermore, different tissues have different expression profiles of HIF-1α, thus the same polymorphism may play different role in different tissues [30,31]. However, all those results should be treated with caution as there were only 2 or 3 studies included in some cancer site subgroups, which might reduce the results’ reliability.

As for subgroup of ethnicity, despite the possible genetic background difference among different ethnicities [32], as well as environment and life-style difference between Asian and Caucasian, A allele of G1790A was found to be significantly associated with increased cancer risk in both Asian and Caucasian population. However, population of this subgroup analysis was limited to Caucasian and Asian only. No study based on African participants found. Further case–control studies with large sample size and multiple ethnicities are required.

It has been reported that HIF-1α may facilitate lymph node metastasis by reducing cell adhesion, degrading extracellular matrix through up-regulating matrix metalloproteinase (MMPs) or lysyl oxydase (LOX) [33] and increasing chemotaxis [3,34]. As G1790A enhances the stability and trans-activating capacity of HIF-1α [12], it is possible that it may also affect the risk of lymph node metastasis. In the current study, the association between A allele carriers (AA or AG) and the risk of lymph node metastasis in 7 case–control studies was not observed. After stratification by ethnicity and control source, significant association of AA or AG genotype with risk of lymph node metastasis was found in Caucasian and Asian, and PB population. However, only 1 or 2 studies were available for each subgroup analysis and only the dominant model (AA + AG vs. GG) was used. Therefore, the results should be treated with caution.

In this study, significant heterogeneity was found in four genetic models in both overall and subgroup analysis. Six studies included in our meta-analysis did not follow HWE. Deviation from HWE reflects potential errors existing in those 6 studies, such as laboratory or genotyping errors, population stratification or selection bias in the choice of controls and unaccounted confounding factors [25,35]. Sensitivity analysis was performed to assess the stability of the current analysis. After excluding those 6 studies deviated from HWE, the conclusions didn’t change, indicating that our conclusions were stable.

There were still some limitations in the current meta-analysis. The sample size for some subgroup analysis was limited, which could increase the possibility of type I and type II errors. Even though our funnel plot and Egger’s test did not show any bias, publication bias was also not avoidable, as positive results have higher chances to be published. Also, most studies were based on Asian and Caucasian population, and no studies were from Africans. Thus, further studies are needed in other ethnic population because of possible ethnic differences of G1790A polymorphism.

## Conclusions

Our meta-analysis suggests that the substitution of G allele with A of HIF-1α gene G1790A polymorphism is a risk factor of cancer, especially for pancreatic cancer, lung cancer, renal cell carcinoma and head and neck cancer. The association is significant in Asian, Caucasion population and public based control subgroups. However, it’s not associated with risk of lymph node metastasis by overall meta-analysis. When stratified by ethnicity and control source, significant higher risk of lymph node metastasis was found in Caucasian, Asian and public based population.

## Competing interests

The authors declare that they have no competing interests.

## Authors’ contributions

Conceived and designed the experiments: YZ, LL, JW, QC and JL Performed the experiments: YZ, LL, YW, XJ, XZ (Xin Zhao), DL, TH, LJ, HD, XZ (Xin Zeng), JL, JW and QC. Analyzed the data: YZ, LL, YW, XJ, XZ (Xin Zhao), DL, TH, LJ, HD, XZ (Xin Zeng), JL, JW and QC. Contributed reagents/materials/analysis tools: YZ, LL, YW, XJ, XZ (Xin Zhao), DL, TH, LJ, HD, XZ (Xin Zeng), JL, JW and QC. Wrote the manuscript: YZ, LL, YW, XJ, XZ (Xin Zhao), DL, TH, LJ, HD, XZ (Xin Zeng), JL, JW and QC. All authors read and approved the final manuscript.
